# Atopic diseases in pediatric population: prematurity and small for gestational age

**DOI:** 10.1186/s12887-025-06380-3

**Published:** 2025-12-13

**Authors:** Yi-Yu Su, Chi-Jen Chen, Mei-Huei Chen, Ching-Chun Lin, Chin-Kan Chan, Wu-Shiun Hsieh, Hsi Chang, Chung-Ming Chen, Hsiu-Chen Lin, Pau-Chung Chen

**Affiliations:** 1 Institute of Environmental and Occupational Health Sciences, College of Public Health, National Taiwan University, Room 733, 17 Xu-Zhou Road, Taipei, 10055 Taiwan; 2 Department of Pediatrics, Taipei Medical University Hospital, Taipei, Taiwan; 3 Institute of Epidemiology and Preventive Medicine, National Taiwan University College of Public Health, Taipei, Taiwan; 4 Institute of Population Health Sciences, National Health Research Institutes, Miaoli, Taiwan; 5 Department of Pediatrics, National Taiwan University College of Medicine and Hospital, Taipei, Taiwan; 6 Department of Pediatrics, Taoyuan General Hospital, Taoyuan, Taiwan; 7 Department of Biotechnology, Ming Chuan University, Taoyuan, Taiwan; 8 Department of Pediatrics, Cathay General Hospital, Taipei, Taiwan; 9 Department of Pediatrics, School of Medicine, College of Medicine, Taipei Medical University, Taipei, Taiwan; 10 Department of Clinical Pathology, Taipei Medical University Hospital, Taipei, Taiwan; 11 Department of Environmental and Occupational Medicine, National Taiwan University College of Medicine and Hospital, Taipei, Taiwan; 12 National Institute of Environmental Health Sciences, National Health Research Institutes, Miaoli, Taiwan; 13 Department of Public Health, National Taiwan University College of Public Health, Taipei, Taiwan

**Keywords:** Asthma, Atopic dermatitis, Allergic rhinitis, Prematurity, Small for gestational age

## Abstract

**Background:**

Atopic diseases, including asthma, atopic dermatitis (AD), allergic rhinitis (AR), and food allergy, are significant chronic conditions in the pediatric population. Prematurity and small-for-gestational-age (SGA) status are critical factors influencing long-term health outcomes. This study investigated the associations between prematurity, SGA, and the development of atopic diseases in children using a nationwide longitudinal cohort.

**Methods:**

We analyzed data from Taiwan’s National Health Insurance Research Database (NHIRD), which includes nearly all residents. The cohort comprised infants born between January 1, 2004, and December 31, 2019, excluding those with early death and multiple births. Premature or SGA infants were designated as study cases, while term, appropriate-for-gestational-age (AGA) infants served as controls. Kaplan-Meier analysis estimated cumulative incidence, and log-rank tests compared disease risk across groups. Cox proportional hazards models, adjusted for demographics, pregnancy-related factors, socioeconomic status, and urbanization, were used to calculate hazard ratios (HRs) with 95% confidence intervals (CIs).

**Results:**

A total of 1,758,460 infants (914,713 males; 843,747 females) were included. Prematurity was associated with increased risks of AR (HR, 1.03) and asthma (HR, 1.19 in males; HR, 1.17 in females) but a lower risk of AD (HR, 0.94 in males; HR, 0.95 in females) in the AGA group. SGA was not associated with atopic diseases in term infants.

**Conclusion:**

Prematurity was linked to higher risks of asthma and AR and a lower risk of AD, while SGA status showed no association with atopic diseases in term infants. Further studies are needed to clarify underlying mechanisms and assess causality.

**Supplementary Information:**

The online version contains supplementary material available at 10.1186/s12887-025-06380-3.

## Introduction

Atopic diseases including asthma, atopic dermatitis (AD), allergic rhinitis (AR), and food allergy, are significant chronic conditions in pediatric population and have shown increasing incidence over the past 30 to 40 years [1]. Despite their lifelong impact on patients and the substantial economic burden they place on healthcare systems, prevention strategies and treatable causes for these conditions remain unclear [2]. Early-life exposures, particularly during prenatal and early childhood periods when the immune system is developing, are thought to play a pivotal role in the pathogenesis of atopic diseases [3]. Research in recent decades has revealed that these conditions often follow a time-based progression, beginning with AD and food allergy in infancy, followed by asthma and AR in later childhood [4]. This phenomenon, known as the “atopic march,” is likely driven by shared genetic and environmental risk factors that shape immune responses across different atopic conditions [5]. While the mechanisms underlying this progression remain incompletely understood, certain risk factors appear to be consistently associated with multiple atopic diseases.

Among these risk factors, prematurity and small-for-gestational-age (SGA) status have been increasingly recognized as key determinants of long-term health outcomes, including the development of atopic diseases [6, 7]. The Developmental Origins of Health and Disease (DOHaD) theory suggests that fetal growth patterns and early developmental exposures may predispose individuals to chronic conditions later in life [8]. Studies indicate that preterm or SGA infants may exhibit distinct patterns of atopic disease development compared to their term or appropriate-for-gestational-age (AGA) counterparts. Notably, prematurity has been associated with an increased risk of asthma but a lower risk of AD, suggesting a differential immune trajectory in preterm infants [6, 9, 10]. In contrast, the relationship between SGA and asthma risk remains inconsistent, with studies reporting positive, null, or even inverse associations [11, 12]. These discrepancies may stem from differences in study populations, sample sizes, and definitions of atopic outcomes.

Given these uncertainties, a large-scale, longitudinal analysis is needed to better understand the relationship between prematurity, SGA status, and the development of atopic diseases. Therefore, we conducted a nationwide cohort study to estimate the association between prematurity, SGA status, and the risk of atopic diseases in the pediatric population.

## Methods

This study utilized data from Taiwan’s National Health Insurance Research Database (NHIRD), which has collected comprehensive medical records for nearly 23 million residents since 1996 and covers 99.9% of the population [13]. In 2015, the Health and Welfare Data Center (HWDC) of the Ministry of Health and Welfare (MOHW) integrated NHIRD with other health-related databases to establish the Taiwan Maternal and Child Health Database (TMCHD), providing reliable mother-child linkages for perinatal and pediatric research [14].

The study cohort included all live births in Taiwan between January 1, 2004, and December 31, 2019. Multiple births were excluded, and infants who died during follow-up were censored from the analysis. Multiple births were excluded because they significantly increase the risk of both prematurity and being born SGA [15]. Excluding them reduced heterogeneity in perinatal conditions and strengthened the validity of our analyses. Prematurity was defined as birth before 37 weeks of gestation, while SGA was defined as birth weight below the 10th percentile for gestational age, as assessed by healthcare providers. Infants were categorized into four groups based on gestational age and birth weight: term AGA (control group), term SGA, preterm AGA, and preterm SGA. Given the well-documented sex-related differences in growth, metabolism, and atopic disease development, all analyses were performed separately for males and females [16, 17].

Diagnoses of asthma, AD, AR, and food allergy were identified using the International Classification of Diseases (ICD) coding system, including both the Ninth and Tenth Revision Clinical Modification (ICD-9-CM and ICD-10-CM) codes. Asthma (ICD-9: 493, ICD-10: J45), atopic dermatitis (ICD-9: 691.8, ICD-10: L20), allergic rhinitis (ICD-9: 477, ICD-10: J30), and food allergy (ICD-9: 693.1, 995.6, ICD-10: Z91.01) were identified using their respective ICD codes. To ensure diagnostic accuracy, atopic disease diagnoses—including asthma, AD, AR, and food allergy—were confirmed based on the requirement of at least three outpatient visits or one hospital admission.

Covariates included in the analysis were demographics (sex, age), pregnancy-related factors (maternal age, pregnancy complications including gestational hypertension, preeclampsia, and premature rupture of membranes), and complications of prematurity (chronic lung disease, cerebral palsy, hydrocephalus, and necrotizing enterocolitis). Socioeconomic status (SES) was assessed using monthly salary data from NHIRD, categorized into three income brackets: <15,840 New Taiwan Dollars (NTD), 15,841–25,000 NTD, and ≥ 25,001 NTD. These thresholds were selected based on Taiwan’s statutory minimum wage (15,840 NTD) during the study period and an additional cutoff at 25,000 NTD to distinguish middle-income earners from higher-income groups. Urbanization level was classified into four categories (Level I: most urbanized to Level IV: least urbanized), based on residential characteristics including population density, educational attainment, the proportion of elderly residents, agricultural employment rates, and access to healthcare resources [18]. Both socioeconomic status and urbanization level were included as covariates in logistic regression and Cox proportional hazards models to adjust for their potential confounding effects.

Descriptive analyses were performed using univariate and bivariate statistics to summarize baseline characteristics and assess initial associations. Cox proportional hazards models were employed to estimate hazard ratios (HRs) and 95% confidence intervals (CIs), adjusting for all specified covariates. The Kaplan-Meier method was used to estimate the cumulative incidence for each atopic disease, and the log-rank test was applied to compare differences among groups. All statistical analyses were conducted using SAS statistical software (version 9.4; SAS Institute, Cary, NC).

## Results

A total of 1,758,460 infants were included in the study, comprising 914,713 males and 843,747 females, born between 2004 and 2019. Infants who died during follow-up were censored, and multiple births were excluded. Table 1 presents the demographic characteristics of the study cohort. The mean gestational age for term AGA infants was 38.6 ± 1 weeks in males and 38.7 ± 1.1 weeks in females, while for term SGA infants, it was 38.8 ± 1 weeks in males and 38.9 ± 1 weeks in females, showing minimal differences from the control group. Preterm AGA infants had a mean gestational age of 34.8 ± 2 weeks in males and 34.8 ± 2.1 weeks in females, whereas preterm SGA infants were born earlier at 34.6 ± 2 weeks in males and 34.5 ± 2.1 weeks in females. Birth weight followed a similar pattern, with term AGA infants having the highest mean birth weights (3192.9 ± 272.8 g in males, 3091.1 ± 265.4 g in females) and preterm SGA infants the lowest (1851.5 ± 404 g in males, 1734.2 ± 401.1 g in females).

**Table 1:** Characteristics of the cohort

Characteristics	Term AGA	Term SGA	Preterm AGA	Preterm SGA
Male				
n	764369	81599	61104	7641
Birth gestational age (weeks)	38.6 (1)	38.8 (1)	34.8 (2)	34.6 (2)
Birth body weight (grams)	3192.9 (272.8)	2602.2 (206.5)	2524.1 (462.1)	1851.5 (404)
Pregnancy related variables				
Maternal age	30.1 (4.7)	29.3 (5)	30.4 (5.2)	30.7 (5.3)
Diabetes	5771 (0.76%)	516 (0.63%)	995 (1.63%)	169 (2.21%)
Hypertension	3546 (0.46%)	1006 (1.23%)	1093 (1.79%)	773 (10.12%)
Premature rupture of membranes	8698 (1.14%)	897 (1.1%)	4679 (7.66%)	587 (7.68%)
Preeclampsia or eclampsia	1353 (0.18%)	535 (0.66%)	862 (1.41%)	973 (12.73%)
Cesarean section	251752 (32.94%)	22491 (27.56%)	25348 (41.48%)	4205 (55.03%)
Prematurity complications^†^				
All	2222 (0.29%)	474 (0.58%)	1897 (3.1%)	412 (5.39%)
Other variables				
Urbanization level				
I (highest)	218909 (28.64%)	21930 (26.88%)	16389 (26.82%)	2092 (27.38%)
II	251231 (32.87%)	26653 (32.66%)	19953 (32.65%)	2342 (30.65%)
III	144135 (18.86%)	15549 (19.06%)	11672 (19.1%)	1496 (19.58%)
IV (lowest)	150094 (19.64%)	17467 (21.41%)	13090 (21.42%)	1711 (22.39%)
Socioeconomic Status: Monthly Salary (NTD)				
< 15840	95779 (12.53%)	13270 (16.26%)	9746 (15.95%)	1346 (17.62%)
15841–25000	319467 (41.79%)	34334 (42.08%)	25641 (41.96%)	3040 (39.79%)
>=25001	349123 (45.67%)	33995 (41.66%)	25717 (42.09%)	3255 (42.60%)
Female				
n	712395	78864	46731	5757
Birth gestational age (weeks)	38.7 (1.1)	38.9 (1)	34.8 (2.1)	34.5 (2.1)
Birth body weight (grams)	3091.1 (265.4)	2524.8 (199)	2424.5 (468)	1734.2 (401.1)
Pregnancy related variables				
Maternal age	30.1 (4.7)	29.3 (4.9)	30.4 (5.2)	30.9 (5.3)
Diabetes	4925 (0.69%)	446 (0.57%)	812 (1.74%)	120 (2.08%)
Hypertension	3441 (0.48%)	884 (1.12%)	1147 (2.45%)	704 (12.23%)
Premature rupture of membranes	7873 (1.11%)	805 (1.02%)	3740 (8%)	354 (6.15%)
Preeclampsia or eclampsia	1391 (0.2%)	575 (0.73%)	915 (1.96%)	918 (15.95%)
Cesarean section	226426 (31.78%)	20723 (26.28%)	20152 (43.12%)	3482 (60.48%)
Prematurity complications^†^				
All	1478 (0.2%)	356 (0.45%)	1350 (2.89%)	288 (5%)
Other variables				
Urbanization level				
I (highest)	204526 (28.71%)	21414 (27.15%)	12635 (27.04%)	1570 (27.27%)
II	234141 (32.87%)	25716 (32.61%)	15060 (32.23%)	1834 (31.86%)
III	134177 (18.83%)	14921 (18.92%)	8947 (19.15%)	1073 (18.64%)
IV (lowest)	139551 (19.59%)	16813 (21.32%)	10089 (21.59%)	1280 (22.23%)
Socioeconomic Status: Monthly Salary (NTD)				
< 15840	89488 (12.56%)	12480 (15.82%)	7969 (17.05%)	1051 (18.26%)
15841–25000	296481 (41.62%)	33444 (42.41%)	19385 (41.48%)	2277 (39.55%)
>=25001	326426 (45.82%)	32940 (41.77%)	19377 (41.46%)	2429 (42.19%)

† Prematurity complications encompassed chronic lung disease, cerebral palsy, hydrocephalus, and necrotizing enterocolitis

Maternal characteristics varied across groups. Mean maternal age at delivery was similar, but pregnancy-related complications were more prevalent in mothers of preterm infants compared to term AGA controls. Gestational hypertension and preeclampsia were particularly elevated in preterm SGA infants (gestational hypertension: 10.12% in males, 12.23% in females; preeclampsia: 12.73% in males, 15.95% in females), compared to term AGA controls (0.46% in males, 0.48% in females for hypertension; 0.18% in males, 0.2% in females for preeclampsia). Although term SGA infants also had a higher incidence of pregnancy complications than controls, the differences were less pronounced. Cesarean section rates were higher in preterm infants, particularly those born SGA.

Neonatal complications associated with prematurity were most frequent in preterm SGA infants, who had the highest rates of chronic lung disease, cerebral palsy, hydrocephalus, and necrotizing enterocolitis (5.39% in males, 5% in females), followed by preterm AGA infants (3.1% in males, 2.89% in females), term SGA infants (0.58% in males, 0.45% in females), and term AGA controls (0.29% in males, 0.2% in females). Additionally, preterm and SGA infants were more likely to reside in highly urbanized areas and have lower socioeconomic status compared to term AGA infants.

### Atopic diseases and risk associations

The incidence of asthma, AD, and AR differed across groups and sexes, as detailed in Tables 2 and 3. Males had higher overall incidence rates of these atopic diseases than females. The average age at diagnosis varied across different atopic diseases and between sexes. Among children who developed each condition, the median age of diagnosis for asthma, allergic rhinitis, atopic dermatitis, and food allergy is summarized by sex in the supplementary table. Prematurity was significantly associated with an increased risk of asthma (HR: 1.19, 95% CI: 1.17–1.21 in males; HR: 1.17, 95% CI: 1.15–1.20 in females) and AR (HR: 1.03, 95% CI: 1.01–1.04 in males; HR: 1.03, 95% CI: 1.02–1.05 in females). In contrast, prematurity was associated with a lower risk of AD (HR: 0.94, 95% CI: 0.92–0.97 in males; HR: 0.95, 95% CI: 0.93–0.98 in females) among AGA infants. Similar patterns were observed in preterm SGA infants, who exhibited increased risks of asthma (HR: 1.17, 95% CI: 1.13–1.22 in males; HR: 1.21, 95% CI: 1.16–1.28 in females) and AR (HR: 1.07, 95% CI: 1.03–1.10 in males; HR: 1.06, 95% CI: 1.02–1.11 in females), but a reduced risk of AD (HR: 0.92, 95% CI: 0.86–0.98 in males; HR: 0.95, 95% CI: 0.88–1.03 in females).

**Table 2:** Allergic diseases in male infants across each group

	Incidence (per 10^5^)	95%CI	Crude HR	95%CI	*P* value	Adjusted HR^†^	95%CI	*P* value
	Asthma							
Term AGA	3287.8	3274.4 To 3301.1	1.00	-		1.00	-	
Term SGA	3274	3233.3 To 3314.7	0.99	0.98 to 1.01	0.3284	1.01	0.99 to 1.02	0.4624
Preterm AGA	3947.7	3894.9 To 4000.5	1.20	1.18 to 1.22	< 0.0001	1.19	1.17 to 1.21	< 0.0001
Preterm SGA	3994.2	3843 To 4145.5	1.20	1.15 to 1.24	< 0.0001	1.16	1.12 to 1.21	< 0.0001
	Atopic dermatitis							
Term AGA	1251.8	1244.1 To 1259.5	1.00	-		1.00	-	
Term SGA	1180.9	1158 To 1203.8	0.95	0.93 to 0.96	< 0.0001	0.98	0.96 to 1.00	0.0181
Preterm AGA	1173.4	1147.1 To 1199.8	0.94	0.92 to 0.96	< 0.0001	0.94	0.92 to 0.97	< 0.0001
Preterm SGA	1159.7	1085.3 To 1234.2	0.92	0.86 to 0.98	0.0107	0.92	0.86 to 0.98	0.0088
	Allergic rhinitis							
Term AGA	5971.9	5952.7 To 5991.1	1.00	-		1.00	-	
Term SGA	5780.3	5722.9 To 5837.7	0.97	0.96 to 0.98	< 0.0001	0.99	0.98 to 1.00	0.1266
Preterm AGA	6144.3	6075 To 6213.5	1.03	1.02 to 1.04	< 0.0001	1.03	1.01 to 1.04	< 0.0001
Preterm SGA	6492.7	6289 To 6696.4	1.08	1.05 to 1.12	< 0.0001	1.06	1.02 to 1.09	0.0012
	Food allergy							
Term AGA	5.9	5.4 to 6.4	1.00	-		1.00	-	
Term SGA	7.2	5.5 to 8.9	1.21	0.94 to 1.56	0.1326	1.18	0.91 to 1.51	0.2119
Preterm AGA	5.7	4 to 7.5	0.98	0.71 to 1.34	0.8816	0.94	0.68 to 1.30	0.6888
Preterm SGA	6.8	1.4 to 12.3	1.15	0.51 to 2.56	0.7386	0.95	0.41 to 2.21	0.9118

† Adjusted for age, pregnancy related variables, prematurity complications and other variables mentioned in Table 1

**Table 3:** Allergic diseases in female infants across each group

	Incidence (per 10^5^)	95%CI	Crude HR	95%CI	*P* value	Adjusted HR^†^	95%CI	*P* value
	Asthma							
Term AGA	2466.2	2454.5 To 2477.8	1.00	-		1.00	-	
Term SGA	2485.6	2450.4 To 2520.7	1.01	0.99 to 1.02	0.4064	1.02	1.00 to 1.03	0.0492
Preterm AGA	2938.9	2888.5 To 2989.4	1.19	1.17 to 1.21	< 0.0001	1.17	1.15 to 1.20	< 0.0001
Preterm SGA	3134.4	2984.4 To 3284.4	1.25	1.19 to 1.31	< 0.0001	1.20	1.14 to 1.26	< 0.0001
	Atopic dermatitis							
Term AGA	1101	1093.6 To 1108.5	1.00	-		1.00	-	
Term SGA	1051.4	1029.6 To 1073.3	0.96	0.94 to 0.98	< 0.0001	0.99	0.96 to 1.01	0.1866
Preterm AGA	1042.2	1014 To 1070.4	0.95	0.92 to 0.98	0.0003	0.95	0.93 to 0.98	0.0006
Preterm SGA	1084.3	1001.4 To 1167.2	0.98	0.90 to 1.05	0.5289	0.95	0.88 to 1.03	0.2295
	Allergic rhinitis							
Term AGA	4577.2	4560.4 To 4594	1.00	-		1.00	-	
Term SGA	4510.1	4460.2 To 4560	0.98	0.97 to 1.00	0.0083	1.00	0.99 to 1.02	0.4855
Preterm AGA	4751.4	4684.3 To 4818.6	1.04	1.03 to 1.06	< 0.0001	1.03	1.02 to 1.05	< 0.0001
Preterm SGA	5006.1	4807.7 To 5204.5	1.09	1.05 to 1.13	< 0.0001	1.05	1.01 to 1.09	0.0186
	Food allergy							
Term AGA	4.5	4 to 4.9	1.00	-		1.00	-	
Term SGA	6.1	4.5 to 7.7	1.37	1.04 to 1.82	0.0272	1.33	1.01 to 1.77	0.0455
Preterm AGA	5.7	3.7 to 7.7	1.28	0.89 to 1.85	0.1865	1.10	0.75 to 1.61	0.6160
Preterm SGA	4.5	0.9 to 13.3	1.01	0.32 to 3.15	0.9866	0.73	0.22 to 2.36	0.5949

† Adjusted for age, pregnancy related variables, prematurity complications and other variables mentioned in Table 1

SGA status in term infants was not associated with increased atopic disease risk. However, in preterm infants, SGA showed a slight but non-significant increase in AR risk compared to preterm AGA infants. Neither prematurity nor SGA was linked to food allergy development during follow-up.

### Cumulative incidence of atopic diseases

**Fig. 1:**
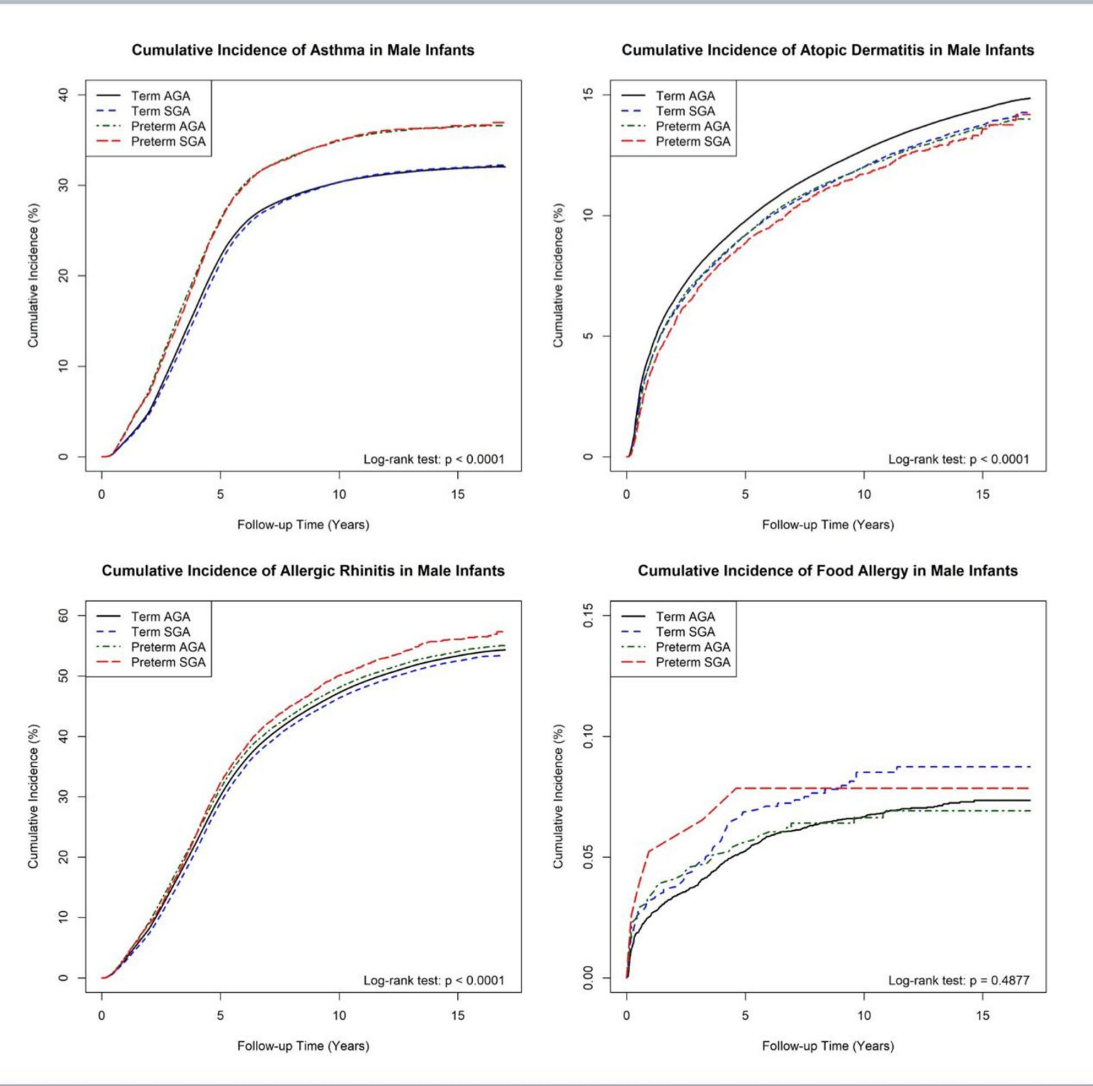
Cumulative incidence of each atopic disease in male infants. Kaplan-Meier survival curves showing the cumulative incidence of asthma, atopic dermatitis (AD), allergic rhinitis (AR), and food allergy in male infants

**Fig. 2:**
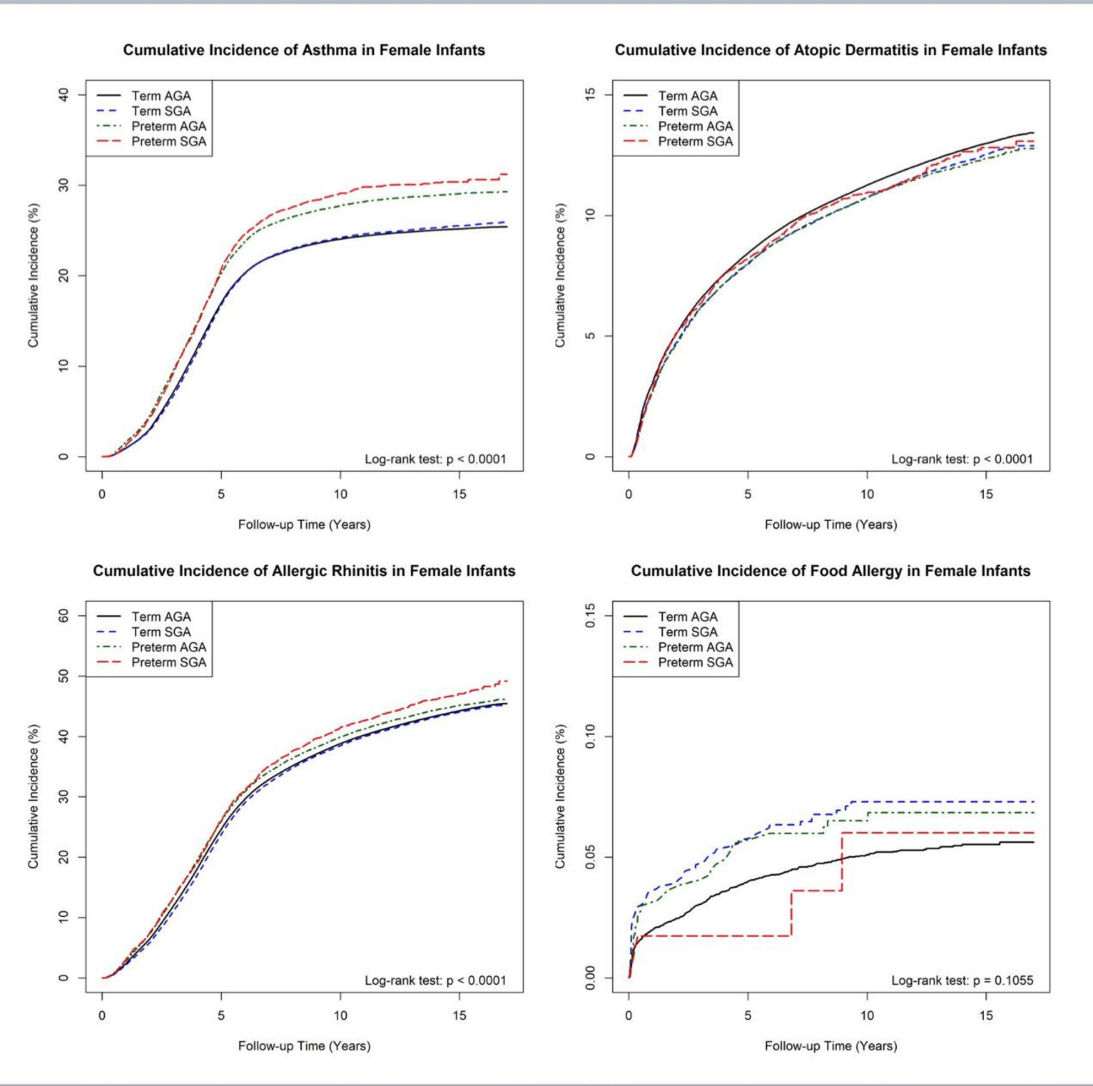
Cumulative incidence of each atopic disease in female infants. Kaplan-Meier survival curves showing the cumulative incidence of asthma, atopic dermatitis (AD), allergic rhinitis (AR), and food allergy in female infants

Figures 1 and 2 present the cumulative incidence curves for atopic diseases across study groups. AD was primarily diagnosed before two years of age, as indicated by the steepest rise in cumulative incidence early in life. Term AGA infants consistently had the highest cumulative incidence of AD throughout the follow-up period.

Asthma diagnoses peaked between two and five years of age, after which the cumulative incidence curve flattened. Preterm infants had a higher cumulative incidence of asthma than term infants, with the largest gap emerging after age five. Among preterm infants, preterm SGA had the highest cumulative incidence of asthma, followed by preterm AGA, term SGA, and term AGA controls.

The incidence of AR followed a similar trend, with most diagnoses occurring between two and five years of age. Preterm infants had higher cumulative incidence of AR than term AGA controls, with preterm SGA infants showing the highest risk.

For food allergy, the highest cumulative incidence was observed in term SGA infants, though the small number of cases suggests that this finding should be interpreted with caution.

## Discussion

### Prematurity and atopic diseases

In this study, prematurity was associated with an increased risk of asthma and AR in childhood but a decreased risk of AD. These findings align with results from other nationwide databases and systematic reviews, which demonstrate a stronger association between prematurity and atopic diseases as gestational age decreases [10, 19–21]. Although the mechanisms underlying prematurity-associated asthma are beyond the scope of this study, multiple contributing factors have been proposed. These include underdeveloped airways, increased antibiotic exposure, heightened susceptibility to viral infections, reduced breastfeeding rates, and an altered gut microbiota [22]. Conversely, the association between prematurity and a lower risk of AD may be linked to increased skin permeability in preterm infants, early antigen exposure in the neonatal intensive care unit, and differences in skin microbiota composition [21]. Preterm infants are born with a thinner stratum corneum and fewer cornified layers; although postnatal skin maturation accelerates after birth, elevated transepidermal water loss often persists until around 34 to 35 weeks of gestation [23]. This compromised skin barrier may lead to earlier and more frequent antigen exposure, potentially inducing immune tolerance through the activation of regulatory T cells and tolerogenic dendritic cells [24]. In contrast, term infants, with a more functionally intact barrier, may encounter environmental antigens later in infancy, which may favor Th2-skewed sensitization and increase the risk of AD. Experimental studies suggest that the timing of antigen exposure is a critical determinant of immune outcomes, with early exposure promoting tolerance and delayed exposure favoring allergic sensitization [25].

The relationship between prematurity and AR remains controversial in the literature. Some studies suggest that prematurity increases AR risk during childhood but may become a protective factor in adulthood [26, 27]. One possible explanation for these inconsistencies is the varying follow-up durations across studies. Atopic diseases often manifest at different stages of childhood, with environmental exposures and immune maturation playing a role in disease development. Over time, medical interventions such as vaccination, inhalers, allergy medications, and immunotherapy, as well as evolving environmental exposures, may modify disease risk. Additionally, asthma and AR share common inflammatory pathways, as evidenced by eosinophilic infiltration in the bronchial mucosa of AR patients, even in the absence of asthma [28]. The “one airway, one disease” concept further supports the close relationship between these two conditions, reinforcing the importance of simultaneous evaluation and treatment [29].

### SGA and atopic diseases

SGA status showed no significant association with atopic diseases in term infants, and only a weak, non-significant association with AR in preterm infants. Prior studies examining fetal growth restriction and atopic diseases have reported conflicting results. For example, a large cohort study from Denmark, Sweden, and Finland found that SGA was associated with a slightly increased risk of asthma-related hospitalization in term infants, whereas a UK-based cohort linked prenatal growth faltering to atopic eczema development in infancy [11, 30]. However, these associations were modest despite large sample sizes and were inconsistent with findings from other research [31].

One potential reason for these discrepancies is the heterogeneous nature of SGA. Fetal growth restriction can manifest as asymmetrical intrauterine growth restriction (IUGR)—often caused by extrinsic factors such as placental insufficiency—or symmetrical IUGR, which may result from intrinsic factors such as chromosomal abnormalities or early intrauterine infections. These varying etiologies may influence immune development differently, complicating the relationship between SGA and atopic diseases. Additionally, IUGR infants are frequently born prematurely, often before SGA status can be fully assessed, which may further obscure associations [32].

Maternal complications such as preeclampsia and gestational hypertension, both strongly linked to fetal growth restriction [33, 34], have also been associated with increased atopic disease risk in offspring [35, 36]. In this study, mothers of SGA infants—especially preterm SGA infants—had higher rates of preeclampsia and gestational hypertension, which may have influenced disease risk despite statistical adjustments. Furthermore, maternal atopic history, a key hereditary risk factor for atopic diseases, is often associated with increased risks of both prematurity and fetal growth restriction [37]. This interplay between maternal health, fetal growth, and atopic disease risk suggests that SGA alone may not be a sufficient marker for developmental exposures influencing atopic disease outcomes.

Although prematurity itself is not a modifiable risk factor, several early-life interventions may help mitigate the risk of allergic diseases in this vulnerable population. These include promoting breastfeeding, early and appropriate introduction of complementary foods, enhancement of the skin barrier through regular emollient use in high-risk neonates, and probiotic supplementation [2, 38, 39]. In addition, environmental strategies such as reducing exposure to indoor and outdoor air pollutants may also be beneficial [40]. These approaches align with emerging evidence favoring microbial diversity and immune training over allergen avoidance. Implementing such preventive measures, along with early screening in high-risk groups like preterm infants, may offer meaningful improvements in long-term atopic outcomes.

### Strengths and limitations

This study has several strengths. It is the first nationwide cohort study in Taiwan to examine the relationship between preterm birth, SGA status, and atopic diseases, leveraging data from a highly representative healthcare system. The large sample size enhances statistical power, and the robust linkage between maternal and child health records enables a comprehensive analysis of perinatal risk factors. The findings provide valuable insights into early-life risk factors for atopic diseases, supporting the development of prevention and screening strategies.

However, certain limitations should be acknowledged. First, some potential confounders, including the severity of SGA, maternal atopic history, and specific environmental exposures, were not available in the dataset, which may have influenced the observed associations. Although limited maternal health data are accessible, atopic diagnoses in adults often lack consistent information on timing, severity, and clinical progression, which limits their utility in this context. Several other known risk factors for childhood atopic diseases, such as maternal smoking exposure, maternal weight, breastfeeding, and family history of atopy, were not available in the NHIRD and therefore could not be included in the analysis. Although viral infections and antibiotic use may be present in claims data, their ubiquity in infancy and close association with medical care in preterm infants complicate meaningful interpretation. The absence of these variables may contribute to residual confounding and should be addressed in future research using more detailed clinical or cohort datasets. Second, our disease definitions requiring at least three outpatient visits or one hospitalization may underestimate the true incidence, as some mild or self-managed cases, particularly those treated through traditional Chinese medicine or over-the-counter remedies, may not be captured in the claims data. Third, the database does not account for participants’ mobility or activity patterns, limiting our ability to assess actual environmental exposures beyond urbanization level. Lastly, While the study covers the full pediatric period, the follow-up duration may be insufficient to fully capture long-term disease progression, particularly for children born in more recent years who had shorter follow-up time. As a result, we did not perform age-specific incidence analyses or stratification by attained age. Future research should explore the age-dependent effects of prematurity and SGA on the development of atopic diseases, including the potential evolution of risk across childhood in relation to the atopic march. As children grow older, additional environmental exposures and lifestyle factors may further influence the associations between early-life risk factors and atopic outcomes.

Previous studies suggest that maternal fish oil supplementation may reduce early-life allergic sensitization and the severity of AD, though the long-term clinical significance remains uncertain [41, 42]. Given Taiwan’s high seafood consumption and potentially higher omega-3 intake, this may represent an unmeasured confounder. However, based on current evidence, its impact on our results is likely limited.

## Conclusion

This study suggests that prematurity is associated with an increased risk of asthma and AR and a reduced risk of AD in Taiwanese children. These findings highlight the need for integrated prevention and early screening strategies tailored to preterm infants. In contrast, SGA was not significantly associated with atopic diseases in term infants, though it may modestly increase the risk of AR in preterm infants. Future research should explore alternative fetal growth indicators, such as the Ponderal Index, to better understand the complex interplay between fetal growth and immune system development. Further investigations are needed to elucidate underlying mechanisms and establish causal relationships between perinatal factors and atopic disease risk. To our knowledge, this is the first large-scale, nationwide cohort study in an Asian population to simultaneously examine prematurity and SGA in relation to multiple atopic outcomes across the pediatric age range. The findings contribute novel insights into early-life risk stratification for allergy prevention in clinical and public health settings.

## Supplementary Information

Supplementary Material 1.

## Data Availability

The datasets analyzed during the current study were obtained from the National Health Insurance Research Database (NHIRD), maintained by the Bureau of National Health Insurance, Department of Health, Taiwan. Due to licensing restrictions, these data are not publicly available. However, they may be accessed upon reasonable request and with appropriate permissions from the National Health Research Institutes (NHRI). Further inquiries can be directed to the corresponding author.
