# Supplementary material for: Atopic diseases in pediatric population: prematurity and small for gestational age
Source: BMC Pediatr. 2025 Dec 13;26:50. doi: 10.1186/s12887-025-06380-3 (PMC12821825; doi:10.1186/s12887-025-06380-3)
Supplement: Supplementary file 1 — Supplementary Material 1. [file 12887_2025_6380_MOESM1_ESM.docx]

Supplementary Table. The age of diagnosis for asthma, allergic rhinitis, atopic dermatitis, and food allergy by sex

| Disease | Median (25^th^ – 75^th^ centiles) age – males | Median (25^th^ – 75^th^ centiles) age – females |
| --- | --- | --- |
| Asthma | 3.76 (2.45 - 5.16) | 3.99 (2.72 - 5.26) |
| Allergic rhinitis | 4.21 (2.57 - 6.12) | 4.28 (2.65 - 6.11) |
| Atopic dermatitis | 2.08 (0.68 - 5.17) | 2.51 (0.96 - 5.49) |
| Food allergy | 2.12 (0.32 - 4.61) | 2.14 (0.22 - 4.61) |
